# Providing Pneumococcal Vaccines Reduces the Risk of Serious Infections From Pneumococcal Pneumonia. We Should Consider a Simplified Immunization Schedule

**DOI:** 10.1093/crocol/otad056

**Published:** 2023-10-27

**Authors:** Mazen Almasry, Freddy Caldera

**Affiliations:** Department of Medicine, University of Wisconsin School of Medicine & Public Health, Madison, WI, USA; Division of Gastroenterology and Hepatology, University of Wisconsin–Madison School of Medicine and Public Health, Madison, WI, USA

Patients with inflammatory bowel disease (IBD) are at an increased risk for several infections, including vaccine-preventable diseases such as influenza and *Streptococcus Pneumonia*. Patients with IBD on certain immune-modifying therapies may be at an increased risk for infections.^1^ Furthermore, infection-related complications are one of the primary causes of mortality among patients with IBD.^2^ Compared to patients hospitalized for other reasons, patients with IBD hospitalized due to a severe infection are at higher risk for mortality.^2^ Higher infection-related mortality has been reported in Crohn’s disease (CD) and ulcerative colitis (hazard ratio [HR] = 3.23, confidence interval [CI]: 2.64–3.94 and HR = 2.21, CI: 1.93–2.53, respectively).^2^ Pneumococcal pneumonia is one of the most common infection-related complications leading to hospitalization among patients with IBD.^3^ A large cohort study involving 108 604 patients with IBD demonstrated an increased risk of pneumonia in patients with IBD as compared to the general population (HR = 1.54, CI: 1.49–1.60).^4^ The risk is particularly pronounced in patients receiving corticosteroids or antitumor necrosis factor (TNF) agents.^4^ Another nationwide cohort study found that the overall rate of pneumonia development in IBD patients was 3.2 per 1000 patient-years. Moreover, the overall hospitalization and mortality rates due to pneumonia ranged from 0.09 to 1.12 per 1000 patient-years.^5^ Given this increased risk, it is recommended that individuals with IBD, especially those on immune-modifying therapy, receive a pneumococcal vaccine.^6^ Despite this recommendation, vaccination rates remain suboptimal.^7^

In this volume of *Crohn’s & Colitis 360*, Ford et al. conducted a retrospective cohort study using TriNetX (Cambridge, MA), a multi-institutional database containing data from over 70 million patients between 2002 and 2022.^8^ The study aimed to assess the risk of hospitalization, intensive care unit (ICU) admission, mechanical ventilation, and/or mortality related to *Streptococcal pneumonia* infection among patients with IBD. They identified 221 957 adult patients with IBD, of whom only 15% were vaccinated vaccination (defined as recorded PCV13, recorded PPSV23, or encounter for pneumococcal vaccine administration). Younger patients, under 50 years of age, were less likely to be vaccinated than older patients who were 60 years of age or older. However, younger patients were vaccinated more frequently if they had a history of advanced therapy prescriptions (immunomodulators, anti-TNF therapy, non-TNF biologics, or Janus kinase inhibitors). Most patients (72%) diagnosed with a severe pneumococcal infection (requiring hospitalization) were unvaccinated. After propensity score matching, vaccinated patients had lower odds of hospitalization (odds ratio [OR] 0.71, 95% CI 0.67–0.76), ICU admission (OR 0.85, 95% CI 0.77–0.93), mechanical ventilation (OR 0.84, 95% CI 0.73–0.97), and mortality (0.65, 95% CI 0.56–0.76) within 30 days of pneumococcal infection.

This study confirms the suboptimal vaccination rates in patients with IBD, with only 15% receiving a pneumococcal vaccine.^8^ The Advisory Committee on Immunization Practices (ACIP) has recommended both PCV 13 and PPSV23 for immunocompromised adults since 2011. Although Ford et al.^8^ defined pneumococcal vaccination as the receipt of either PCV13 or PPSV23, vaccination rates were still lower than reported rates in a recent systematic review, estimated at 20%.^7^ Notably, older age was highlighted among positive vaccine predictors in this study. In a recent systemic review summarizing the literature on predictors of pneumococcal vaccination, the use of immunosuppressive therapy, older age, and healthcare professionals’ recommendations were positive predictors of pneumococcal vaccination.^7^ While the authors of this study^8^ included IBD-specific medication prescriptions as a factor in defining IBD to increase sensitivity, it limited the ability to assess the impact of immunosuppressive therapy on infection-related complications. Several studies have shown that anti-TNF therapy may blunt the immune response against the pneumococcal vaccine, which is a significant concern. A meta-analysis conducted by Muller et al.^9^ showed no significant difference in the seroconversion rates after pneumococcal vaccine between patients with IBD on immunosuppressive monotherapy and those without immunosuppressive treatment. However, subanalysis of the data revealed that anti-TNF therapy was associated with lower seroconversion rates to pneumococcal vaccination compared to patients not on immunosuppressive treatment.^9^ Furthermore, this analysis was limited since it only evaluates the immunogenicity of a pneumococcal vaccine in patients on thiopurines and/or anti-TNF biologic therapy. There is a paucity of data evaluating the immunogenicity of vaccines in patients on non-TNF biologic (ustekinumab, vedolizumab, or risankizumab) and small molecule therapy such as Janus kinase inhibitors.

The findings of this study provide evidence that pneumococcal vaccination reduces the risk of infection-related complications, including mortality.^8^ However, vaccine uptake remains suboptimal. A recent systemic review exploring vaccine hesitancy in patients with IBD identified concerns about vaccination side effects and misconceptions about vaccination as the primary reasons.^7^ Reassuring evidence is available as a meta-analysis study confirmed that vaccines, including pneumococcal, are safe and well tolerated, with no reported hospitalizations or deaths following immunization. Additionally, vaccinations do not result in a substantial increase in IBD flares.^10^ Conversely, a different study identified the lack of pneumococcal vaccination recommendations by healthcare providers as a significant barrier.^11^ The need for consensus regarding whether IBD providers or primary care physicians should be responsible for immunization is a primary reason for missing essential vaccinations.^1^ Given the hesitancy by both primary care physicians and gastroenterologists in taking ownership of vaccinations, the American College of Gastroenterology preventive care IBD guidelines suggest that gastroenterology providers should share the responsibility for vaccination recommendations. They should own the responsibility for recommending vaccines and share the responsibility for administering vaccines.^1^ Therefore, it is recommended that gastroenterologists share equal responsibility with primary care physicians in ensuring that patients are appropriately vaccinated. Ideally, vaccines could be administered by gastroenterologists when patients during clinic visits. Strategies outlined by Bhat et al. can aid the successful implementation of vaccination programs in outpatient gastroenterology practices.^12^ These strategies may involve identifying a “vaccine champion” who educates healthcare providers and collaborates with pharmacy departments to manage vaccine inventory. Furthermore, improving healthcare providers’ education was highlighted as a primary intervention to enhance the pneumococcal vaccination rate.^7^

**Figure 1.**
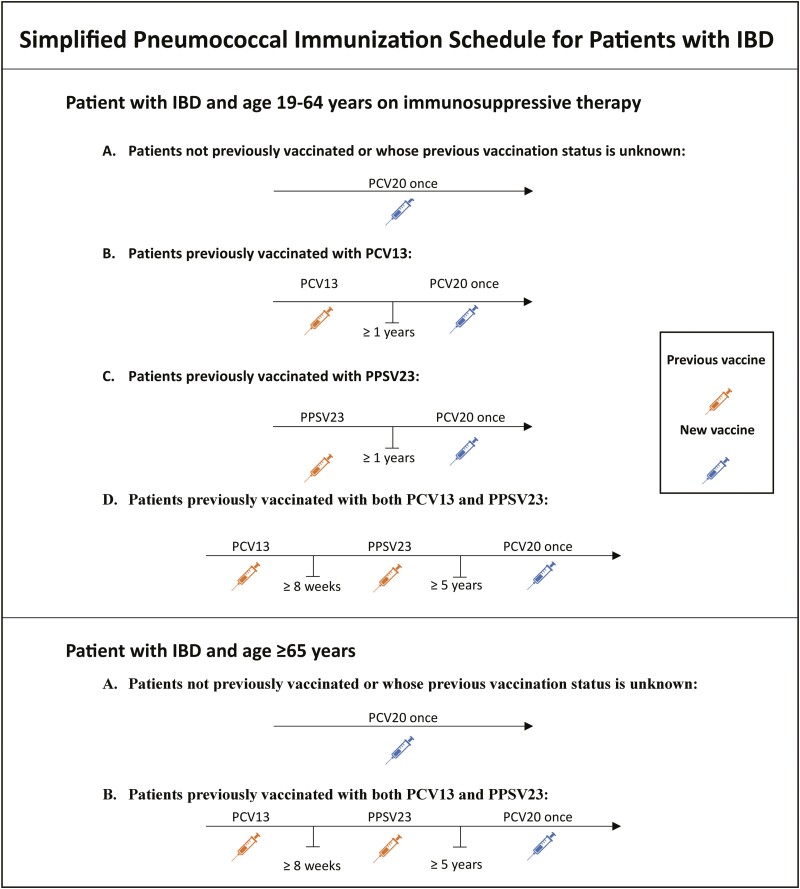
Simplified pneumococcal immunization schedule for patients with IBD based on the Advisory Committee on Immunization Practices (ACIP) recommendations. IBD, inflammatory bowel disease; PCV20, pneumococcal 20-valent conjugate vaccine; PCV13, pneumococcal 13-valent conjugate vaccine; PPSV23, pneumococcal polysaccharide vaccine.

The ACIP has recently issued new recommendations for two novel pneumococcal vaccines (PCV 20 and PCV 15) both with a broader serotype spectrum.^6^ No studies have evaluated the immunogenicity of PCV 20 or PCV15 in immunosuppressed populations. Given that there is no preference for one vaccine, we propose using PCV 20 for patients with IBD since only 1 dose is needed in all situations (Figure 1). The simplified PCV20 vaccination schedule of just 1 dose may improve vaccination uptake among patients with IBD.^6^

In summary, the study by Ford et al.^8^ highlights the suboptimal vaccination rates in patients with IBD despite the vaccine’s effectiveness in protecting against invasive pneumococcal disease. These findings call for implementing system-based programs to improve pneumococcal vaccination rates in patients with IBD. We suggest focusing on PCV 20 to simplify the pneumococcal immunization schedule. Further studies are needed to evaluate the rates and efficacy of the new pneumococcal vaccines.
